# No. 11 Blade‐Based Surgical Techniques for Steatocystoma Multiplex: A Systematic Review

**DOI:** 10.1111/jocd.71021

**Published:** 2026-07-02

**Authors:** Jia Qi Adam Bai, Sherilyn Wen, Aleksandra Frątczak, Chaocheng Liu

**Affiliations:** ^1^ Faculty of Medicine University of Ottawa Ottawa Ontario Canada; ^2^ Temerty Faculty of Medicine University of Toronto Toronto Ontario Canada; ^3^ Department and Clinic Dermatology, Faculty of Medical Sciences in Katowice Medical University of Silesia Katowice Poland; ^4^ Department of Dermatology and Skin Science University of British Columbia Vancouver British Columbia Canada; ^5^ School of Medicine Simon Fraser University Surrey British Columbia Canada

**Keywords:** minimal incision technique, no. 11 blade, steatocystoma multiplex, systematic review

To the Editor,

Steatocystoma multiplex (SM) is a rare benign disorder of the pilosebaceous unit characterized by the development of multiple sebum‐filled dermal cysts that commonly occur on the trunk, neck, and proximal extremities and often represent a cosmetic concern [1]. Recently, simple minimally invasive surgical approaches using a No. 11 blade to create a small incision followed by expression and removal of the cyst wall have increasingly been reported as a promising therapy with favorable cosmetic outcomes. This systematic review aimed to evaluate the efficacy and safety of No. 11 blade‐based surgical techniques for the treatment of SM.

This systematic review followed PRISMA guidelines and was prospectively registered on PROSPERO (CRD420261293468). MEDLINE and Embase were searched with predefined keywords (Table S1). Evidence quality was assessed using the Grading of Recommendations, Assessment, Development, and Evaluation approach. Articles were independently screened by two reviewers, resulting in six articles reporting on 53 patients (Figure 1; Table 1) [2, 3, 4, 5, 6, 7].

**FIGURE 1:**
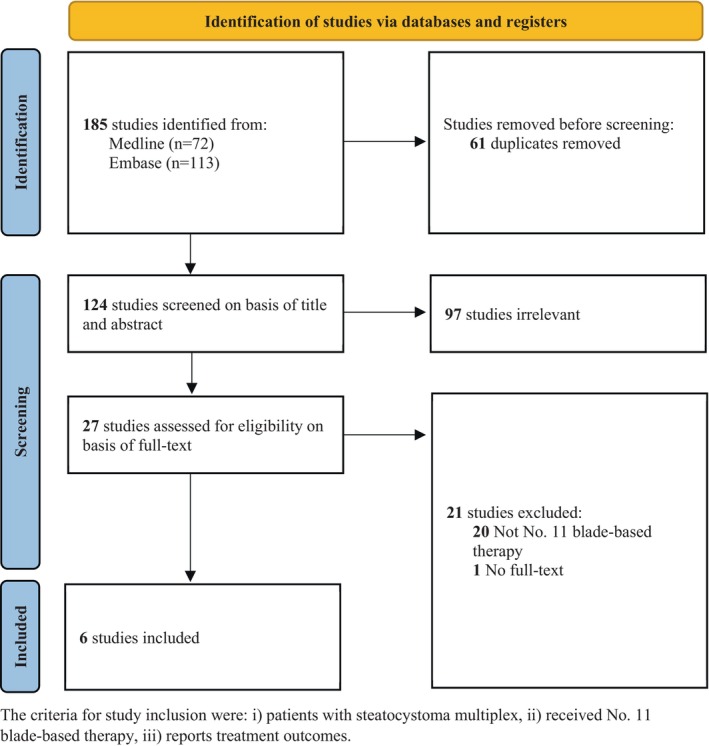
Flow diagram of literature screening using the Preferred Reporting Items for Systematic Reviews and Meta‐Analyses (PRISMA) guidelines. Figure adapted from http://prisma‐statement.org.

**TABLE 1:** Characteristics, certainty of evidence, and relevant clinical outcomes for steatocystoma multiplex patients treated with No. 11 blade therapy.

Study (Author, year) [GRADE]	Study type	*N* patients	Mean age	Sex	Lesions per patient	Lesion site (n)	Lesion size (n)	Treatment summary	Post‐op care	Outcome (n)	Therapy duration	Follow‐up (months)	Scar formation (*n*)	Patient satisfaction
MacKenzie, 2025 [2] [Low]	CAR	1	37	F (1)	45	Labia majora (45)	1–5 mm (45)	Local anesthesia, incised cysts with N11B, applied forceps to target area, performed curette debridement and cautery	Wound dressing	CR (1)	One‐time	6	None (1)	High
Jiang, 2021 [3] [Moderate]	PCS	40	24.8	M (22), F (18)	NR	Neck (NR), trunk (NR), upper extremities (NR)	NR	Local anesthesia, incised cysts with N11B, used forceps to pull cysts out	Tissue adhesive, wound cleaning	CR (40)	One‐time	12	Minimal (40)	High
Lee, 2007 [4] [Low]	CAS	1 of 5	53	M (1)	12	Neck (12)	5–10 mm (12)	Local anesthesia, incised cysts with N11B, used vein hook to grasp the cyst, used forceps to pull it out	Sterile strips, wound cleaning	CR (1)	One‐time	22	Minimal (1)	NR
Lee, 2007 [4] [Low]	CAS	2 of 5	28	M (1)	14	Arm (14)	3–5 mm (14)	NR	12	CR (1)	One‐time	15	Minimal (1)	NR
Lee, 2007 [4] [Low]	CAS	3 of 5	24	M (1)	11	Forearm (11)	4–6 mm (11)	NR	6	CR (1)	One‐time	30	Minimal (1)	NR
Lee, 2007 [4] [Low]	CAS	4 of 5	31	F (1)	8	Neck (8)	5–7 mm (8)			CR (1)	One‐time	19	Minimal (1)	NR
Lee, 2007 [4] [Low]	CAS	5 of 5	46	M (1)	5	Face (5)	7–10 mm (5)			CR (1)	One‐time	14	Minimal (1)	NR
Lee, 2005 [5] [Low]	CAR	1	33	F (1)	8	Arms (NR), chest (NR)	5–15 mm (8)	Local anesthesia, incised cysts with N11B, extirpated cyst walls with forceps	NR	CR (1)	One‐time	6	None (1)	NR
Schmook, 2001 [6] [Low]	CAR	5	NR	NR	NR	NR	NR	Incised cysts with N11B, excochleated cyst walls with curet, removed cysts with forceps	NR	CR (5)	One‐time	NR	None (5)	NR
Adams, 1999 [7] [Low]	CAR	1	27	M (1)	NR	Chest (NR), neck (NR)	NR	Incised cysts with N11B, removed cysts with forceps	NR	CR (1)	One‐time	4	None (1)	NR

Abbreviations: CAR, case report; CAS, case series; CM, centimeters; CR, complete response; F, female; M, male; MM, millimeters; N11B, no. 11 blade; NR, not reported; PCS, prospective cohort study.

Of the 53 patients, the mean age was 26.5 years with 56.3% (27/48) males (Table 1). Mean number of lesions per patient was 14.7 (range: 5–45) and lesion size ranged from 1 to 15 mm. Lesions most commonly involved the genitalia (47.4%, 45/95), upper extremities (26.3%, 25/95), and neck (21.1%, 20/95).

All six included studies employed a minimally invasive incision technique using a No. 11 blade followed by expression of cyst contents and removal of cyst wall using adjunctive instruments such as forceps (100%, 6/6), curettes (33.3%, 2/6), or vein hooks (16.7%, 1/6). Procedures were completed in a single treatment session (100%, 6/6) and were typically performed under local anesthesia (66.7%, 4/6). Postoperative care was variably reported and included simple wound cleaning with alcohol‐soaked gauze (33.3%, 2/6), tissue adhesive closure (16.7%, 1/6), sterile strips (16.7%, 1/6), or wound dressings (16.7%, 1/6).

Complete resolution of treated lesions was reported in all patients (100%, 53/53). Mean follow‐up duration was 12.4 months (range: 4.0–30.0). Cosmetic outcomes were favorable, with minimal scarring described as practically unnoticeable in 81.1% (43/53) of patients, while no scarring was reported in 18.9% (10/53). Transient hyperpigmentation was reported in one study and resolved within 2–4 weeks. When reported, patient satisfaction was described as high (100%, 2/2). No adverse events or recurrences were reported during follow‐up.

SM lesions are composed of cystic structures arising from the pilosebaceous unit, often requiring complete removal of the cyst wall to prevent recurrence [1]. Traditional excisional approaches can be time‐consuming and impractical in patients with numerous lesions. Alternative treatments such as surgical excision, cryotherapy, or laser‐based modalities may also result in suboptimal cosmetic outcomes, often leaving visible scars [8, 9]. No. 11 blade‐based techniques allow creation of small incisions that facilitate expression and extraction of the cyst wall with minimal tissue disruption, enabling treatment of multiple lesions in a single session while preserving cosmetic outcomes [3, 6]. These findings suggest that No. 11 blade‐based techniques represent a simple, safe, and effective approach for the treatment of SM. Study limitations include the small number of included studies, heterogeneity in treatment protocols, the low level of evidence inherent to case reports and case series, incomplete reporting of patient and procedural characteristics across studies, and the potential for reporting bias. Larger prospective studies with standardized treatment protocols are warranted to better define the efficacy, recurrence, and cosmetic outcomes of No. 11 blade‐based techniques for SM.

## Funding

The authors did not receive any specific funding for this study.

## Ethics Statement

An ethics statement is not applicable because this study is based exclusively on published literature.

## Consent

The authors have nothing to report.

## Conflicts of Interest

The authors declare no conflicts of interest.

## Supporting information


**Table S1:** Search strategy used for literature screening.

## Data Availability

The data underlying this article are available in the article and in its Supporting Information.
