# Supplementary material for: No. 11 Blade‐Based Surgical Techniques for Steatocystoma Multiplex: A Systematic Review
Source: J Cosmet Dermatol. 2026 Jul 2;25(7):e71021. doi: 10.1111/jocd.71021 (PMC13328807; doi:10.1111/jocd.71021)
Supplement: Supplementary file 1 — Table S1: Search strategy used for literature screening. [file JOCD-25-e71021-s001.docx]

**Supplemental Table 1.** Search strategy used for literature screening.

Ovid MEDLINE(R) ALL <1946 to January 29, 2026>

| **#** | **Search line** | **Results** |
| --- | --- | --- |
| 1 | exp Steatocystoma Multiplex/ | 80 |
| 2 | "steatocystoma*".ab,kf,ti. | 332 |
| 3 | steatocystoma multiplex.ab,kf,ti. | 260 |
| 4 | exp Surgical Instruments/ | 27175 |
| 5 | (scalpel* or blade* or "No. 11" or "No. 11 blade" or "number 11" or "11 blade").ab,kf,ti. | 22378 |
| 6 | (incision* or surg* or excis* or extract* or expression or curett* or excochleat*).ab,kf,ti. | 6620573 |
| 7 | 1 or 2 or 3 | 359 |
| 8 | 4 or 5 or 6 | 6647241 |
| 9 | 7 and 8 | 72 |

Embase Classic+Embase <1947 to 2026 January 29>

| **#** | **Search line** | **Results** |
| --- | --- | --- |
| 1 | exp Steatocystoma Multiplex/ | 220 |
| 2 | "steatocystoma*".ab,kf,ti. | 428 |
| 3 | steatocystoma multiplex.ab,kf,ti. | 323 |
| 4 | exp Surgical Instruments/ | 761455 |
| 5 | (scalpel* or blade* or "No. 11" or "No. 11 blade" or "number 11" or "11 blade").ab,kf,ti. | 29740 |
| 6 | (incision* or surg* or excis* or extract* or expression or curett* or excochleat*).ab,kf,ti. | 9072819 |
| 7 | 1 or 2 or 3 | 484 |
| 8 | 4 or 5 or 6 | 9492750 |
| 9 | 7 and 8 | 113 |
